# Oral appliance therapy for obstructive sleep apnea: a retrospective study in a psychiatric sleep clinic

**DOI:** 10.20407/fmj.2022-023

**Published:** 2022-12-27

**Authors:** Kota Funahashi, Marina Hirose, Suguru Kondo, Yoshimi Sano, Shiho Fujita, Nakao Iwata, Tsuyoshi Kitajima

**Affiliations:** 1 Department of Psychiatry, Fujita Health University, School of Medicine, Toyoake, Aichi, Japan; 2 Department of Plastic and Reconstructive Surgery, Division of Pediatric Dentistry and Orthodontics, Fujita Health University, School of Medicine, Toyoake, Aichi, Japan; 3 Department of Laboratory Medicine, Fujita Health University Hospital, Toyoake, Aichi, Japan

**Keywords:** Obstructive sleep apnea, Oral appliance, Discontinuation, Sleepiness, Psychiatric disorder

## Abstract

**Objectives:**

We evaluated the continuity and effectiveness of oral appliances (OAs) for treating obstructive sleep apnea (OSA) in a psychiatric sleep clinic, specifically focusing on mild cases and those with psychiatric comorbidity.

**Methods:**

We retrospectively examined the medical records of 106 OSA patients treated with OA. Survival analysis was performed to assess the discontinuation of OA use. Clinical Global Impression-Improvement (CGI-I) scale were obtained from medical records. The apnea-hypopnea index (AHI), measured by polysomnography (PSG), and Epworth Sleepiness Scale (ESS) were compared between diagnosis and after post-OA treatment if a second PSG for efficacy assessment was conducted.

**Results:**

Among all 106 patients, Kaplan-Meier analysis estimated a discontinuation rate of 16.8% at 1 year. This tended to be higher for OSA patients with psychiatric comorbidity (22.7%) than those without (11.6%), though it was not statistically significant (*P*=0.08). The overall rate of improvement in CGI-I scale was 37.7% and was significantly lower in OSA patients with psychiatric comorbidity (25.0%) than those without (48.3%). Among the 74 patients who underwent a second PSG, AHI and ESS were significantly lower after OA treatment for the entire group and subgroups of OSA severity at diagnosis and psychiatric comorbidity, except for ESS in the moderate OSA severity subgroup.

**Conclusion:**

OA continuation was relatively good, and sleepiness was relieved by OA use, even in mild OSA patients and those with psychiatric comorbidity. However, the continuation and subjective improvement of symptoms were slightly lower in OSA patients with psychiatric comorbidity.

## Introduction

Obstructive sleep apnea (OSA) is a sleep disorder in which apnea or hypopnea lasting for ≥10 s occurs repeatedly during sleep.^1^ Many patients awaken feeling tired and unrefreshed and experience excessive daytime sleepiness regardless of sleep length. Associated symptoms include cardiovascular abnormalities, such as hypertension, arrhythmia, and heart failure; metabolic abnormalities, such as impaired glucose tolerance and obesity; and neuropsychiatric problems, such as depression and cognitive dysfunction.

OSA is diagnosed when the apnea-hypopnea index (AHI), which is measured using polysomnography (PSG), is ≥5 in addition to relevant subjective symptoms or certain comorbidity. OSA severity is defined as: mild, 5≤AHI<15; moderate, 15≤AHI<30; and severe, AHI ≥30.^2^ In Japan, continuous persistent air pressure (CPAP) therapy is indicated for the treatment of OSA with AHI ≥20, whereas oral appliance (OA) therapy is indicated for AHI ≥5 and is covered by public insurance. For moderate to severe cases of OSA, CPAP has been shown to improve daytime sleepiness,^3^ blood pressure,^4^ insulin resistance,^5^ and depression.^6^ In psychiatric sleep clinic settings, OSA is often mild and/or comorbid with depression and other psychiatric disorders,^7^ and in such cases, OA therapy is an option to improve sleepiness or other psychosomatic symptoms. Several meta-analyses have shown that OA is effective for daytime sleepiness,^8^ depression,^6^ and hypertension.^9^ However, the benefit of treatments, including OA therapy, in mild OSA cases remains poorly studied,^10^ and the necessity for OA treatment in mild cases is unclear in clinical situations. Furthermore, the continuity and efficacy of OA treatment in cases with psychiatric comorbidity have not been fully clarified.

Therefore, the present retrospective study examined the continuity and effectiveness of OA treatment for OSA patients in a psychiatric sleep clinic, with a particular focus on the differences between mild and moderate cases and between those with and without psychiatric comorbidity.

## Methods

### Participants

Data were collected retrospectively from the medical records of patients who had visited the Department of Psychiatry, Fujita Health University Hospital, Japan, and were treated with OA following a diagnosis of OSA from May 1, 2005, to November 30, 2019. Diagnosis and treatment were made at the discretion of the attending physician within the scope of usual practice. Only cases in which PSG was performed in our psychiatry department and those who had OAs created by dentists at our hospital were included in the analysis. If OAs were created more than once for the same participant, only the data for the first OA were used. The present study was approved by the ethics committee of Fujita Health University (HM21-057). An opt-out procedure was used, and individual patient consent was not required because of the retrospective nature of the study.

### Data collection

OSA was diagnosed by the attending physician and confirmed retrospectively based on the International Classification of Sleep Disorders, Third edition.^1^ Clinical information (age, sex, body mass index, and comorbid psychiatric disorders) was obtained from medical records. PSG data were collected for all cases at the time of diagnosis, and for those who underwent a second PSG for efficacy assessment, the self-rated Epworth Sleepiness Scale (ESS)^11^ score was obtained at the time of PSG. The Clinical Global Impression-Improvement (CGI-I) scale^12^ was used by two psychiatrists (including one certificated physician of the Japanese Society of Sleep Research) to evaluate sleep-related conditions according to the descriptions of the attending physicians immediately after the completion of OA, 3–9 months after OA use was deemed stable, and after 1 year of OA use. The date of the CGI-I evaluation after 1 year to evaluate the final effect of OA therapy was determined by the two psychiatrists as the date of the visit when the sleep-related evaluation was most likely to have been confirmed in the description of medical records (or the date of the visit when discontinuation was confirmed after 1 year). Diagnoses of psychiatric comorbidity made by the attending physician were obtained from medical records.

### OA

**Figure 1:**
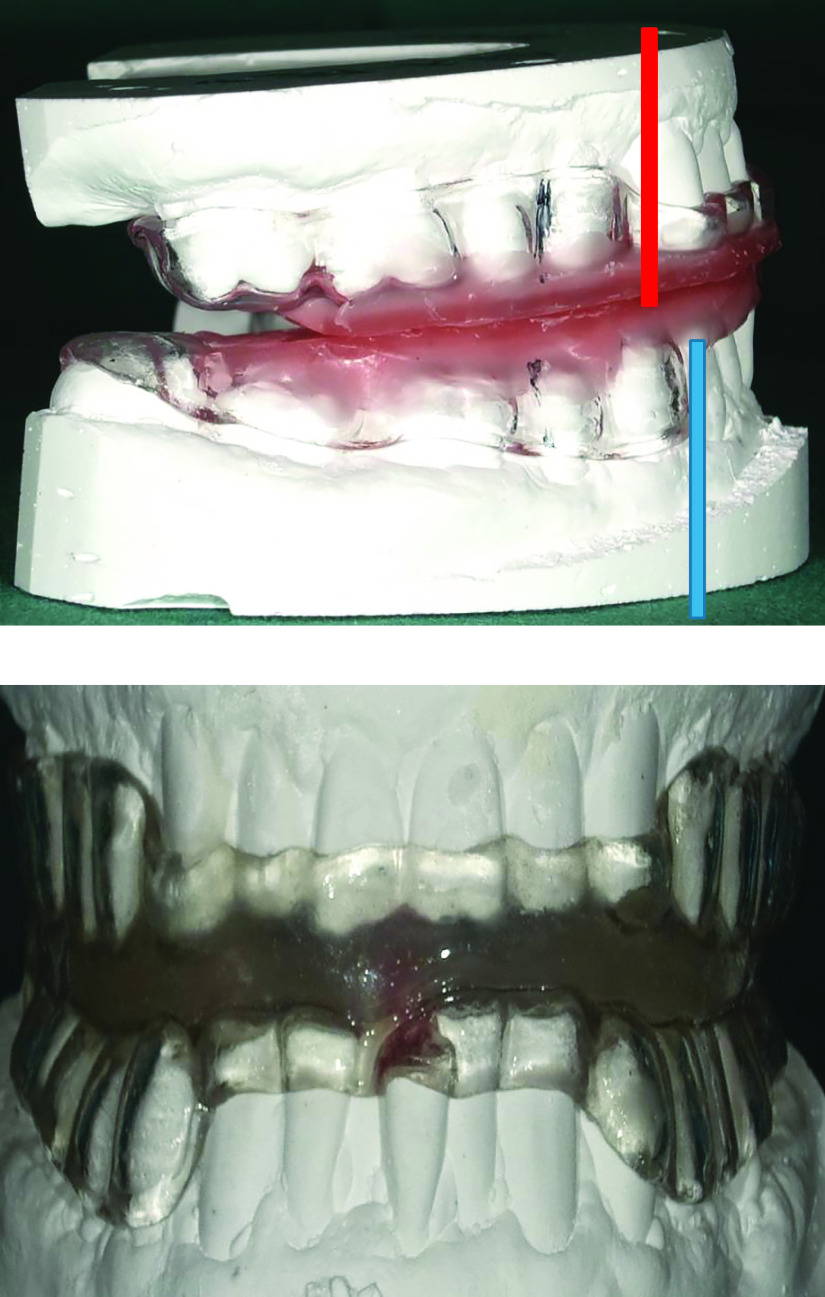
A non-adjustable monoblock mandibular advancement device created at our hospital. The blue horizontal line indicates the advancement of the lower jaw ahead of the upper jaw (red horizontal line).

OAs were created by dentists at Fujita Health University Hospital as non-adjustable monoblock mandibular advancement devices (Figure 1). These were custom-made for individual patients because they are clinically more effective and have higher adherence than those that are ready-made.^13^ We used a non-adjustable device because it is covered by public insurance and is more durable and avoids oral respiration than adjustable devices. The level of mandibular advancement was decided according to the discretion of the attending dentists (approximately 75% of patients’ maximum advancement capability).

### Measurements

The ESS^11^ measures the likelihood of dozing during daily activities. It comprises eight items, each of which has four possible responses (0 to 3). The total score of the items (0 to 24) represents the level of sleepiness, with higher scores indicating greater sleepiness. The ESS is a globally standardized assessment for subjective daytime sleepiness.

The CGI-I^12^ is a rating scale that measures treatment response in clinical studies of patients with psychiatric disorders. Physicians and other clinicians rate patients according to the available clinical information based on their clinical experience. The scale evaluates the degree of overall improvement or exacerbation of a patient’s illness on a seven-point scale from baseline at the start of the intervention: 1: very much improved, 2: much improved, 3: minimally improved, 4: no change, 5: minimally worse, 6: much worse, and 7: very much worse). This scale was used for this study because it was most suitable for retrospectively evaluating the overall effectiveness of OA therapy according to physicians’ descriptions in medical records.

### PSG

PSG was performed according to the manual of the American Academy of Sleep Medicine (AASM)^14^ and included electroencephalogram, electrooculogram (to record eye movement), chin electromyogram (EMG), electrocardiogram, expiration thermistor measurements, chest and abdominal expansion, arterial oxygen saturation, snoring, and leg EMG measurements. PSG parameters were automatically scored using one of four PSG systems (Alice 3 [Respironics, Murrysville, PA, USA], Somnostar Pro [Viasys, Conshohocken, PA, USA], SandmanElite [Covidien, Boulder, CO, USA], or Somnoscreen [Somnomedics, Randersacker, Germany]) and revised manually by trained technicians. Analyses were performed using the Rechtschaffen and Kales criteria^15^ until August 2015, and the 2007 AASM criteria^14^ after September 2015.

### Analysis

The discontinuation rate of OA therapy was calculated using survival analysis, which was performed by identifying the date of the last observation during OA use or the date of confirmation of discontinuation of use according to medical records. The longest final observation date was the CGI-I evaluation date (defined as above) ≥1 year after the start of OA therapy. The date of the final visit was used for patients in whom the visit was interrupted or the final observation occurred within 1 year. Discontinuation was defined as all-cause stoppage of the use of OA, including treatment change to CPAP. The discontinuation rate was estimated using Kaplan-Meier analysis for the entire patient group as well as subgroups according to OSA severity at diagnosis (mild [5≤AHI<15] or moderate [15≤AHI<30]) and the presence or absence of psychiatric comorbidity. In the multivariate Cox proportional hazards model, the hazard ratio of discontinuation was calculated for the severity of OSA at diagnosis or the presence of psychiatric comorbidity by adjusting for sex and age at diagnosis.

For the CGI-I scale score, the endpoint was ≥1 year after OA completion. For patients whose visit was interrupted or the final observation occurred within 1 year, the last CGI-I scale score immediately after the start of OA therapy or after 3–9 months was used and cumulatively added up. The rate of improvement was calculated by assigning patients to two groups: (1) mild improvement or better and (2) unchanged or worsened, plus discontinuation. Rates were compared using χ^2^ tests with Yates’ correction for continuity for OSA severity at diagnosis or presence of psychiatric comorbidity.

AHI, measured by PSG, and simultaneously scored ESS were compared between diagnosis and post-OA treatment for patients who underwent a second PSG for efficacy assessment of OA therapy. For the analysis of PSG data, patients assessed using the Rechtschaffen and Kales^15^ and the 2007 AASM criteria^14^ were treated equally, whereas those assessed using different criteria for the two PSGs were excluded from the analysis. Wilcoxon signed-rank tests were used to compare PSG parameters and ESS. Analyzes were performed for the entire group and separately for subgroups based on OSA severity at diagnosis and the presence of psychiatric comorbidity. Patients with missing data were excluded from the pairwise analysis for the corresponding item or parameter only.

Statistical analyses were performed using EZR 1.55 (open-source software^16^) and JMP 14.2.0 (SAS Institute Japan, Tokyo, Japan). The significance level was set at *P*<0.05.

## Results

### Summary of participants

A total of 106 patients (mean age, 41.1±10.9 [standard deviation (SD) ] years, 87 males) were included in the analysis (Table 1). PSG analysis at diagnosis revealed 35 patients with moderate OSA and 71 with mild OSA. There were 48 patients with psychiatric comorbidities, including depression (n=21), bipolar disorder (n=10), schizophrenia (n=4), anxiety disorder (n=7), obsessive-compulsive disorder (n=3), autism spectrum disorder (n=3), dissociative disorder (n=1), conversion disorder (n=1), adjustment disorder (n=1), cognitive dysfunction (n=1), neurocognitive disorder (n=1), and alcohol dependence (n=1, with overlap), and 58 patients had no psychiatric comorbidity.

### Course of OA treatment

**Figure 2:**
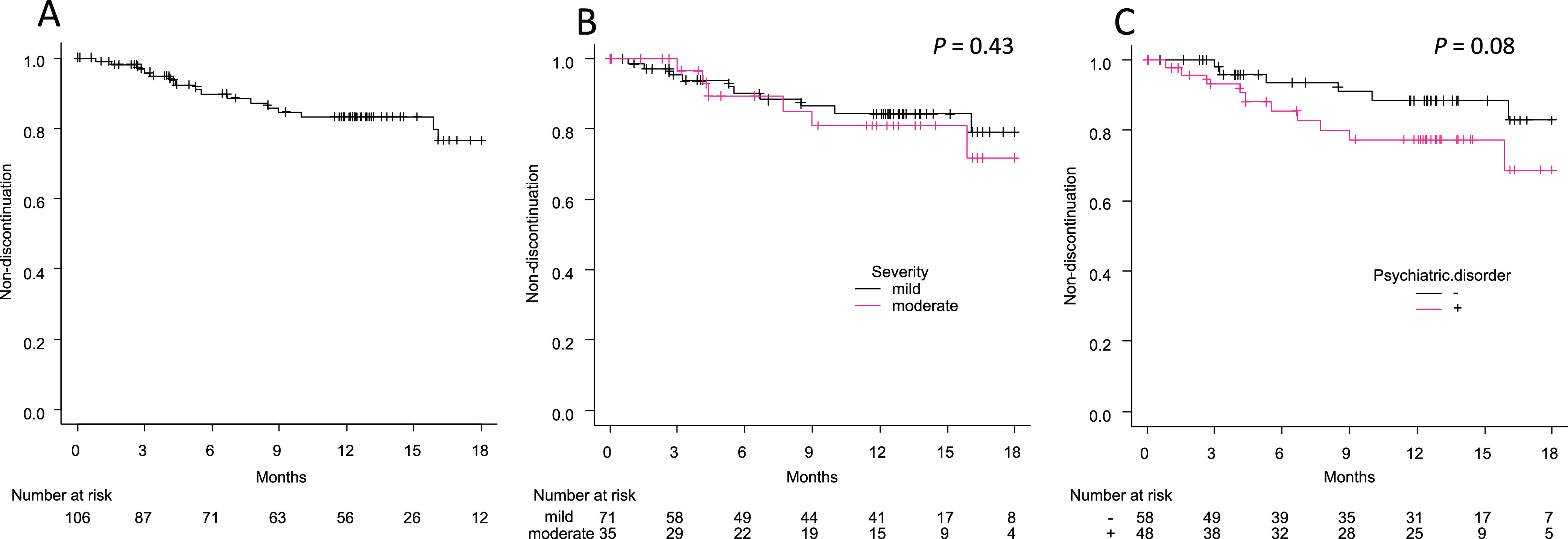
Kaplan-Meier analysis of OA discontinuation. (A) All patients. (B) Severity of OSA at diagnosis (mild vs moderate). (C) Psychiatric comorbidity (with vs without). *P*-values were calculated using log-rank test.

One year after OA completion, 56 patients continued to visit the clinic without clear discontinuation of OA use, 18 discontinued using OA, and 32 had interrupted visits to the clinic. Reasons for discontinuation of OA included CPAP induction (n=5), temporomandibular joint pain (n=5), nausea (n=2), dental treatment (n=1), poor improvement in sleepiness (n=1), poor feeling during use (n=2), and unknown (n=2). Kaplan-Meier analysis showed that the estimated rate of OA discontinuation at 1 year was 16.8% in the entire group (Figure 2A), 15.6% and 19.1% in the mild and moderate OSA groups, respectively (Figure 2B), and 22.7% and 11.6% in the psychiatric comorbidity and no psychiatric comorbidity groups, respectively (Figure 2C). There was no significant difference in OA discontinuation rate according to severity at diagnosis (*P*=0.43), whereas there was a trend difference according to the presence of psychiatric comorbidity (*P*=0.08). The hazard ratios for OA discontinuation adjusted for sex and age were 1.32 for moderate severity and 2.01 for positive psychiatric comorbidity (with no significant association), whereas female sex had a significant risk for discontinuation (Table 2). The explorative analysis using the Kaplan-Meier method stratifying by sex showed that there was a trend of higher OA discontinuation in patients with psychiatric comorbidity than in those without psychiatric comorbidity among male (n=87, *P*=0.05) but not female patients (n=19, *P*=0.80; Supplementary Figure S1).

Among the 56 patients who continued to visit without OA discontinuation, 25 showed mild improvement and 31 showed no change in CGI-I score. When the CGI-I scores before interruption were further cumulated for the 32 patients with interrupted visits within 1 year, the CGI-I scores showed mild improvement or better in 37.7% (40/106) of the entire group, 42.3% (30/71) and 28.6% (10/35) of the mild and moderate OSA groups, respectively, and 25.0% (12/48) and 48.3% (28/58) of the psychiatric comorbidity and no psychiatric comorbidity groups, respectively. There were no significant differences in CGI-I score according to OSA severity at diagnosis (*P*=0.25); however, there was a significant difference according to the presence of psychiatric comorbidity (*P*=0.02).

### Changes in PSG and ESS before and after OA use

Among the 74 patients who underwent a second PSG for efficacy assessment of the OA (mean age, 41.9±12.1 years; 66 males; mean interval between two PSGs, 280.5±199.4 days; Table 3), the AHI showed a significant reduction in the entire group (12.5±5.0 [before] vs 6.6±5.8 [after]; *P*<0.01) as well as in the mild OSA (9.5±2.5 vs 4.9±4.2, *P*<0.01), moderate OSA (18.7±2.3 vs 10.5±7.1, *P*<0.01), psychiatric comorbidity (9.8±4.7 vs 7.7±4.7, *P*<0.01), and no psychiatric comorbidity (12.0±4.7 vs 9.9±5.0, *P*<0.01) groups. ESS was significantly reduced in the entire group (11.1±4.8 vs 9.0±5.0, *P*<0.01) and in the mild OSA (11.3±4.6 vs 9.0±4.9, *P*<0.01), psychiatric comorbidity (9.8±4.7 vs 7.7±4.7, *P*=0.02), and no psychiatric comorbidity (12.0±4.7 vs 9.9±5.0, *P*<0.01) groups. However, ESS showed no significant change in the moderate OSA group (10.7±5.3 vs 9.1±5.3, *P*=0.17).

The PSG parameters before and after OA use are presented in Supplementary Table S1 for the entire group. Supplementary Table S2 shows the PSG parameters according to OSA severity at diagnosis, and Supplementary Table S3 shows the PSG parameters according to the presence of psychiatric comorbidity.

## Discussion

In the present study, Kaplan-Meier analysis estimated that the 1-year OA discontinuation rate was as low as 16.8%. Similar to our findings, Attali et al. reported that long-term use of OA was associated with an estimated 1-year discontinuation rate of approximately 20% using the same method,^17^ although the exact figure was not explicitly stated. A meta-analysis of trials comparing the effects of CPAP and OA in patients with mild to moderate OSA showed higher adherence to OA.^18^ Taken together, these results suggest that sustained use of OA is relatively easy to achieve.

In terms of OSA severity, the estimated discontinuation rate at 1 year was 15.6% in the mild OSA group and 19.1% in the moderate OSA group, which did not differ significantly. OA has been suggested to be less effective than CPAP in improving sleepiness,^18^ and in the present study, several patients with moderate OSA discontinued OA and switched to CPAP.

The estimated discontinuation rate at 1 year for the group with psychiatric comorbidity tended to be higher than for that without psychiatric comorbidity (22.7% and 11.6%, respectively, *P*=0.08). To the best of our knowledge, the present study is the first to report this finding. After adjusting for sex and age, which have been reported to affect OA adherence,^19^ the Cox proportional hazards model showed that the hazard ratio of OA discontinuation with the presence of psychiatric comorbidity was 2.01 (0.73–5.54, *P*=0.18). The discontinuation rate was significantly higher among females in the adjusted covariates, which was an unexpected finding. The association between psychiatric comorbidity and the high discontinuation rate may have been confounded to some extent by the slightly higher proportion of females in the psychiatric comorbidity group. However, the explorative analysis showed that there was a trend of higher discontinuation in male patients with psychiatric comorbidity than without (*P*=0.05), which suggested that psychiatric comorbidity itself had some impact on continuous OA use in males, who have a preponderance of OSA. Among patients with psychiatric comorbidity who discontinued OA, nine had mood disorders, one had adjustment disorder, and one had obsessive-compulsive disorder. Furthermore, the reasons for discontinuation of OA therapy among the patients with psychiatric comorbidity were similar to those without psychiatric comorbidity (data not shown); however, depression and/or low motivation may have hindered regular OA use or affected patients’ tolerance to the stress of continuous OA use. Another possibility is that it was difficult for the patients with psychiatric comorbidity to recognize the effects of treatment outweighing the above problems. Nonetheless, a 77.3% non-discontinuation rate at 1 year is considered relatively favorable. Moreover, the possible sex difference in OA continuation rate warrants further investigation in future studies, especially given that previous studies have yielded conflicting results.^20,21^

The cumulative ratio of mild improvement or better on the CGI-I score after >1 year of OA therapy was relatively low (<50%) when cases with OA discontinuation were taken into account. A possible factor that affected the rate of improvement is the retrospective evaluation of the CGI-I score from medical records by the physicians who conducted the study. Most descriptions in the medical records were based on subjective complaints of patients, which were further subjectively evaluated by the attending physicians at the time of the visit. Therefore, the CGI-I scores were the result of a “triple” subjective evaluation and lacked objectivity. In addition, the descriptions in the medical records were not necessarily based on an adequate sleep-related evaluation performed during each occasion. The good OA continuation rate and the clear improvements in the AHI and ESS indicate that a specifically defined or objective evaluation could have revealed an even greater clinical improvement. Furthermore, a relatively large proportion of patients had psychiatric comorbidities that were associated with particularly low rates of improvement as measured by the CGI-I scale, which may have contributed to the lower overall rate of improvement.

The percentage of patients that showed an improvement in CGI-I score tended to be lower in those with moderate OSA severity than in those with mild OSA severity, although this did not reach significance. Some patients with moderate OSA severity switched from OA to CPAP, which suggested relatively insufficient efficacy of OA in this group. In terms of psychiatric comorbidity, the percentage of patients that showed improvement in CGI-I score was significantly lower in those with psychiatric comorbidity than in those with no psychiatric comorbidity. Specifically, patients with mood disorders may have had symptoms such as insomnia and midnight awakening, and OA alone may not have been sufficient to improve these symptoms in patients who were concurrently undergoing treatment for psychiatric comorbidities.

In patients who underwent a second PSG for efficacy assessment, AHI and ESS significantly decreased in response to OA use in most cases, including those with mild OSA severity at diagnosis and psychiatric comorbidity. This suggests that OA may also be an effective treatment for OSA in these groups. However, because this analysis was only performed in patients who were able to complete the second PSG, the continued use of OA itself may indicate that a certain level of efficacy had been achieved. In terms of severity, there was a decreasing trend in ESS in moderate OSA cases, although this was not statistically significant. This may be partly because there were only 35 moderate OSA cases, which was fewer than the number of mild OSA cases and may have led to insufficient statistical power. However, it is also possible that the beneficial effect on sleepiness was not as obvious in moderate OSA cases as it was in mild OSA cases. In moderate OSA, it may be more difficult for patients to recognize the therapeutic effect of OA than CPAP; thus, switching to CPAP may appear more effective in some patients with moderate OSA. There was a significant decrease in ESS regardless of the presence or absence of psychiatric comorbidity. In patients with psychiatric comorbidity, improvement in CGI-I was not clear in many cases; however, the results suggest that OA may improve sleepiness, which is one of the primary endpoints of the treatment.

The present study has several limitations. First, the number of cases was small, which may have underpowered some of our statistical analyses. In addition, the number of covariates was large compared with the number of events included in the Cox proportional hazards model; thus, overfitting cannot be ruled out. Second, the present study was retrospective, and there could have been issues with data collection. CGI-I score was judged retrospectively by two physicians according to attending physicians’ entries in medical records. These assessments were not controlled using specific evaluation criteria and were indirect evaluations that were not based on patient interviews. In addition, because this was an observational study of routine clinical practice, our observations would have encompassed the effects of treatments other than OA as well as the placebo effect of OA use. In particular, in patients with psychiatric comorbidity, the influences of the comorbidity itself and the medications used may have overlapped. Therefore, rigorous verification of the effects of OA should be assessed prospectively in randomized controlled trials. Third, the rate of OA discontinuation may not be accurate because we did not include a systematic follow-up (e.g., a structured visit or telephone interview following the visit). Specifically, patients who had interrupted visits to the clinic after their OA was created could not be followed for subsequent OA use, and thus, such cases might be regarded as discontinuation of treatment. Nevertheless, in the present study, many patients agreed with their physicians to interrupt visits being satisfied with OA, including those who underwent a second PSG to confirm OA effectiveness. This differs from various other treatments (e.g., pharmacotherapy for psychiatric disorders) because OA can be used continuously without subsequent visits, and the interruption of visits do not necessarily lead to discontinuation of treatment. However, there was also uncertainty around the continuation of OA treatment among those who continued their visits because we determined discontinuation according to patients’ statements, despite the possibility that patients could have discontinued OA use latently. Therefore, the actual discontinuation rate may have been higher on a “patient basis” than estimated. Nevertheless, the discontinuation rate observed is valid on a “clinic basis.”

A strength of this study is that we were able to recruit a sufficient number of patients who showed significant improvement in AHI and ESS across the entire group as well as in the mild OSA severity, psychiatric comorbidity, and no psychiatric comorbidity subgroups. In addition, two physicians performed strict evaluations of the CGI-I score, albeit without specific criteria; thus, there is likely little bias in the evaluations. This allowed the effects of OSA severity and psychiatric comorbidity on OA treatment to be evaluated.

## Conclusion

Analysis of OA therapy for OSA at our psychiatric sleep clinic showed overall good treatment continuation but a trend of slightly higher discontinuation in those with psychiatric comorbidity. OA treatment improved CGI-I score in nearly half of all patients with mild OSA severity or no psychiatric comorbidity; however, the effect was slightly smaller in those with moderate OSA severity or those with psychiatric comorbidity (only the latter reached statistical significance). Significant decreases in AHI and ESS were observed in patients who underwent a second PSG to determine the efficacy of OA. A significant decrease in AHI was observed regardless of OSA severity and the presence of psychiatric comorbidity, whereas a decrease in ESS was not significant in patients with moderate severity. These results suggest that OA may be a worthwhile treatment for patients with mild OSA severity or those with psychiatric comorbidity, particularly to improve sleepiness. However, treatment continuity and subjective improvement may be slightly poorer in patients with psychiatric comorbidity.

## Figures and Tables

**Table1:** Demographic data of all study participants

	All	Severity			Psychiatric Disorder	
		Mild	Moderate		+	−
N	106	71	35		48	58
Sex, Male（%）	87 (82.1)	60（85.0）	27（77.1）		37（77.0）	50（86.2）
Age (years)	41.1±10.9	40.3±9.4	42.9±13.4		40.7±9.0	41.5±12.4
BMI	26.3±4.9	25.8±4.6	27.4±5.5		27.4±5.8	25.4±3.9
Psychiatric Disorder, + (%）	48（45.2）	32 (45.1)	16 (45.7)		—	—
*CGI-I (cummulative)*						
Improved	40	30	10		12	28
No change	48	30	18		25	23
Discontinued	18	11	7		11	7
Obaserved Period (days)	333.1±235.1	341.5±237.3	316.1±232.9		307.0±192.5	354.7±264.9
ESS^#^	11.6±5.0	11.8±4.9	11.3±5.2		10.2±4.7	12.8±5.0
*PSG*						
TIB (min)	486.6±29.4	485.7±29.5	488.5±29.7		489.8±27.3	484.0±31.1
SPT (min)	454.9±45.0	452.8±46.9	459.2±41.1		447.3±50.1	461.2±39.5
TST (min)	391.2±62.0	390.0±68.8	393.5±45.9		375.3±69.3	404.3±52.3
SE (%)	80.5±12.3	80.4±13.5	80.8±9.6		76.8±14.2	83.6±9.6
Wake (%SPT)	14.2±11.3	14.2±11.3	14.1±8.8		16.4±13.3	12.3±9.1
REM (%TST)	16.3±7.3	16.1±7.9	16.7±6.0		14.2±6.9	18.1±7.3
N1/S1 (%TST)	14.4±11.1	13.8±11.6	15.5±10.1		16.0±12.2	13.1±10.1
N2/S2 (%TST)	63.1±12.0	63.4±12.6	62.4±10.9		65.9±11.9	60.7±11.7
N3/S3+S4 (%TST)	6.2±6.9	6.6±7.3	5.3±6.2		3.9±5.4	8.1±7.5
ArI (/hr)	19.7±8.5	17.2±5.5	24.7±11.2		19.7±10.4	19.7±6.7
SL (min)	28.4±34.3	28.6±35.5	27.8±32.2		39.9±42.5	18.8±21.8
REM SL (min)	140±84.5	143.3±87.2	133.6±79.7		159.1±94.8	124.6±72.4
AHI (/hr)	12.9±5.1	10.0±2.8	18.7±3.2		13.2±4.9	12.6±5.2
AI (/hr)	3.6±4.0	2.4±2.3	5.7±5.6		3.5±4.6	3.6±3.5
HI (/hr)	9.3±4.7	7.5±3.1	13.0±5.3		9.7±4.6	9.0±4.8
PLMI(/hr)^#^	5.6±12.6	4.9±10.1	7.2±17.1		6.8±13.9	4.7±24.8

Values are presented as means±standard deviations. There were missing data for the ESS^#^ (n=2 patients) and PLMI^#^ (n=13 patients) calculations. PSG data based on the Rechtschaffen and Kales (R & K) and 2007 AASM criteria were treated equally. ESS, Epworth Sleepiness Scale; PSG, polysomnography; TIB, time in bed; SPT, sleep period time; TST, total sleep time; SE, sleep efficiency; REM, rapid eye movement sleep stage; N1, non-REM stage 1 (AASM); S1, sleep stage 1 (R & K); N2, non-REM stage 2 (AASM); S2, sleep stage 2 (R & K); N3, non-REM stage 3 (AASM); S3, sleep stage 3 (R & K); S4, sleep stage 4 (R & K); SL, sleep latency; ArI, arousal index; HI, hypopnea index; PLMI, periodic limb movement index

**Table2:** Hazard ratios of OA discontinuation

	HR	95%CI	*P*
Severity (Moderate)	1.32	0.50–3.53	0.5731
age^†^	0.96	0.91–1.01	0.1933
sex	3.38	1.26–9.15	0.0154

Psychiatric Disorder (+)	2.01	0.73–5.54	0.18
age^†^	0.96	0.90–1.01	0.185
sex	3.07	1.14–8.27	0.0265

HRs were calculated using multivariate Cox proportional hazard model, and *P*-values were calculated using Wald test. HR for age(†) is shown as a unit risk ratio. HR, hazard ratio; CI, confidence interval

**Table3:** Demographic data of patients who underwent a second PSG to assess the efficacy of OA use

	All	Severity			Psychiatric Disorder	
		Mild	Moderate		+	−
N	74	50	24		29	45
Sex, Male（%）	66 (89.2)	45 (90.0)	21 (87.5)		26 (89.7)	40 (88.9)
Age (years)	41.9±12.1	40.7±10.0	44.4±15.6		41.9±10.3	41.9±13.3
BMI	26.2±4.7	25.7±4.5	27.6±5.0		26.8±5.7	25.9±4.1
Psychiatric Disorder, + (%)	29 (39.2)	21 (42.0)	8 (33.3)		—	—
PSGs Interval (days)	280.5±199.4	291.6±203.8	223.8±93.2		283.0±151.6	261.0±194.0
ESS^#^						
before OA	11.1±4.8	11.3±4.6	10.7±5.3		9.8±4.7	12.0±4.7
after OA	8.9±4.9	8.8±4.8	9.1±5.3		7.7±4.7	9.7±5.0
AHI (/hr)						
before OA	12.5±5.0	9.6±2.5	18.7±2.3		12.6±5.1	12.5±4.9
after OA	6.6±5.8	4.9±4.2	10.1±7.1		7.3±6.4	6.1±5.4

Values are presented as means±standard deviations. There were missing data for the ESS^#^ (n=1 patient), and the corresponding patient was excluded from both of before and after OA use for calculation. PSG, polysomnography; ESS, Epworth Sleepiness Scale; OA, oral appliance
