# Supplementary material for: Oral appliance therapy for obstructive sleep apnea: a retrospective study in a psychiatric sleep clinic
Source: Fujita Med J. 2022 Dec 27;9(3):218–24. doi: 10.20407/fmj.2022-023 (PMC10405904; doi:10.20407/fmj.2022-023)
Supplement: Supplementary file 1 — Supplementary Tables [file fmj-9-218-s001.pdf]

Supplementary Table S1. PSG parameters before and after OA use for the entire group

	All (N = 74)	
	Before OA	After OA
TIB (min)	487.2 ± 30.8	481.9 ± 23.6
SPT (min)	461.6 ± 40.8	458.1 ± 37.7
TST (min)	399.6 ± 57.6	400.9 ± 53.3
SE (%)	82.0 ± 10.9	83.1 ± 10.0
Wake (%SPT)	13.4 ± 10.2	12.6 ± 9.3
REM (%TST)	16.6 ± 7.6	17.2 ± 6.1
N1/S1 (%TST)	13.1 ± 10.9	12.9 ± 9.7
N2/S2 (%TST)	63.7 ± 12.4	63.9 ± 11.2
N3/S3+S4 (%TST)	6.6 ± 6.9	5.9 ± 5.9
ArI (/h)	18.6 ± 6.5	13.9 ± 8.5
SL (min)	22.8 ± 28.5	19.6 ± 22.2
REM SL (min)	137.6 ± 78.9	132.3 ± 86.2
AHI (/h)	12.5 ± 5.0	6.6 ± 5.8
AI (/h)	3.7 ± 3.9	1.3 ± 1.9
HI (/h)	8.9 ± 4.6	5.3 ± 5.0
PLMI(/h)[#]	5.8 ± 12.2	8.2 ± 17.9

Values are presented as means ± standard deviations. There were missing data for the PLMI# (n = 18 patients) calculation.

PSG data were based on the Rechtschaffen and Kales (R & K) and 2007 AASM criteria and were treated equally.

PSG, polysomnography; TIB, time in bed; SPT, sleep period time; TST, total sleep time; SE, sleep efficiency; REM, rapid eye movement sleep stage; N1, non-REM stage 1 (AASM); S1, sleep stage 1 (R & K); N2, non-REM stage 2 (AASM); S2, sleep stage 2 (R & K); N3, non-REM stage 3 (AASM); S3, sleep stage 3 (R & K); S4, sleep stage 4 (R & K); SL, sleep latency; ArI, arousal index; HI, hypopnea index; PLMI, periodic limb movement index

Supplementary Table S2. PSG parameters before and after OA use according to OSA severity at diagnosis

	Mild (N = 50)		Moderalte (N = 24)	
	Before OA	After OA	Before OA	After OA
TIB (min)	485.5 ± 29.2	481.8 ± 16.3	490.7 ± 34.1	482.0 ± 34.6
SPT (min)	460.7 ± 41.4	457.3 ± 36.4	463.5 ± 40.4	459.5 ± 40.1
TST (min)	401.8 ± 62.8	402.0 ± 50.9	395.1 ± 45.7	398.6 ± 59.2
SE (%)	82.8 ± 11.7	83.4 ± 9.8	80.7 ± 9.0	82.7 ± 10.4
Wake (%SPT)	12.9 ± 10.7	12.2 ± 9.5	14.9 ± 8.9	13.5 ± 9.2
REM (%TST)	16.7 ± 8.3	17.8 ± 6.5	16.4 ± 6.1	16.0 ± 5.2
N1/S1 (%TST)	12.6 ± 11.7	11.4 ± 8.7	14.1 ± 9.2	16.1 ± 11.0
N2/S2 (%TST)	63.6 ± 13.3	65.1 ± 11.2	64.0 ± 10.6	61.6 ± 11.1
N3/S3+S4 (%TST)	7.1 ± 7.1	5.8 ± 6.2	5.4 ± 6.4	6.3 ± 5.4
ArI (/h)	16.5 ± 5.2	12.9 ± 8.4	23.0 ± 7.0	20.1 ± 21.8
SL (min)	21.5 ± 30.8	19.3 ± 22.5	25.5 ± 23.4	20.1 ± 21.8
REM SL (min)	133.8 ± 82.3	130.6 ± 92.3	145.1 ± 72.7	136.1 ± 72.9
AHI (/h)	9.6 ± 2.5	4.9 ± 4.2	18.7 ± 2.3	10.1 ± 7.1
AI (/h)	2.6 ± 2.3	0.9 ± 1.2	5.8 ± 5.5	2.0 ± 7.8
HI (/h)	6.9 ± 2.8	4.0 ± 4.0	12.9 ± 4.9	8.0 ± 5.1
PLMI(/h)[#]	4.8 ± 10.1	6.2 ± 12.2	7.9 ± 16.1	13.0 ± 26.7

Values are presented as means ± standard deviations. There were missing data for the PLMI# (n = 18 patients) calculation.

PSG data were based on the Rechtschaffen and Kales (R & K) and 2007 AASM criteria and were treated equally.

PSG, polysomnography; TIB, time in bed; SPT, sleep period time; TST, total sleep time; SE, sleep efficiency; REM, rapid eye movement sleep stage; N1, non-REM stage 1 (AASM); S1, sleep stage 1 (R & K); N2, non-REM stage 2 (AASM); S2, sleep stage 2 (R & K); N3, non-REM stage 3 (AASM); S3, sleep stage 3 (R & K); S4, sleep stage 4 (R & K); SL, sleep latency; ArI, arousal index; HI, hypopnea index; PLMI, periodic limb movement index

Supplementary Table S3. PSG parameters before and after OA use according to the presence of psychiatric comorbidity

	With a psychiatric disorder (N = 29)		No psychiatric disorder (N = 45)	
	Before OA	After OA	Before OA	After OA
TIB (min)	490.4 ± 23.5	484.7 ± 27.1	485.3 ± 34.8	480.1 ± 21.1
SPT (min)	456.8 ± 42.9	459.3 ± 43.5	464.7 ± 39.6	457.2 ± 33.9
TST (min)	385.1 ± 64.1	407.8 ± 56.7	409.0 ± 51.6	396.4 ± 51.2
SE (%)	78.6 ± 12.6	84.1 ± 10.0	84.3 ± 9.1	82.5 ± 10.0
Wake (%SPT)	15.7 ± 11.6	11.7 ± 8.8	11.9 ± 9.0	13.2 ± 9.8
REM (%TST)	13.2 ± 6.9	15.7 ± 7.1	18.8 ± 7.3	18.2 ± 5.2
N1/S1 (%TST)	15.7 ± 11.6	14.3 ± 11.0	11.4 ± 10.3	12.0 ± 8.7
N2/S2 (%TST)	67.4 ± 11.6	65.0 ± 13.4	61.4 ± 12.4	63.3 ± 9.7
N3/S3+S4 (%TST)	3.6 ± 5.0	5.1 ± 6.8	8.5 ± 7.3	6.5 ± 5.3
ArI (/h)	17.5 ± 6.3	13.8 ± 8.8	19.3 ± 7.0	14.0 ± 8.5
SL (min)	31.5 ± 36.6	22.4 ± 24.4	17.1 ± 20.4	22.4 ± 24.4
REM SL (min)	158.9 ± 82.3	160.6 ± 101.6	124.7 ± 74.8	114.7 ± 70.7
AHI (/h)	12.6 ± 5.1	7.3 ± 6.4	12.5 ± 4.9	6.1 ± 5.4
AI (/h)	3.4 ± 4.3	1.5 ± 2.5	3.8 ± 3.7	1.2 ± 1.5
HI (/h)	9.2 ± 4.8	5.8 ± 5.5	8.7 ± 4.5	5.0 ± 4.7
PLMI(/h)[#]	4.6 ± 9.4	7.6 ± 15.4	6.5 ± 13.7	8.6 ± 19.4

Values are presented as means ± standard deviations. There were missing data for the PLMI# (n = 18 patients) calculation.

PSG data were based on the Rechtschaffen and Kales (R & K) and 2007 AASM criteria and were treated equally.

PSG, polysomnography; TIB, time in bed; SPT, sleep period time; TST, total sleep time; SE, sleep efficiency; REM, rapid eye movement sleep stage; N1, non-REM stage 1 (AASM); S1, sleep stage 1 (R & K); N2, non-REM stage 2 (AASM); S2, sleep stage 2 (R & K); N3, non-REM stage 3 (AASM); S3, sleep stage 3 (R & K); S4, sleep stage 4 (R & K); SL, sleep latency; ArI, arousal index; HI, hypopnea index; PLMI, periodic limb movement index
