# Supplementary material for: Oral appliance therapy for obstructive sleep apnea: a retrospective study in a psychiatric sleep clinic
Source: Fujita Med J. 2022 Dec 27;9(3):218–24. doi: 10.20407/fmj.2022-023 (PMC10405904; doi:10.20407/fmj.2022-023)
Supplement: Supplementary file 2 — Supplementary Figure [file fmj-9-218-s002.pdf]

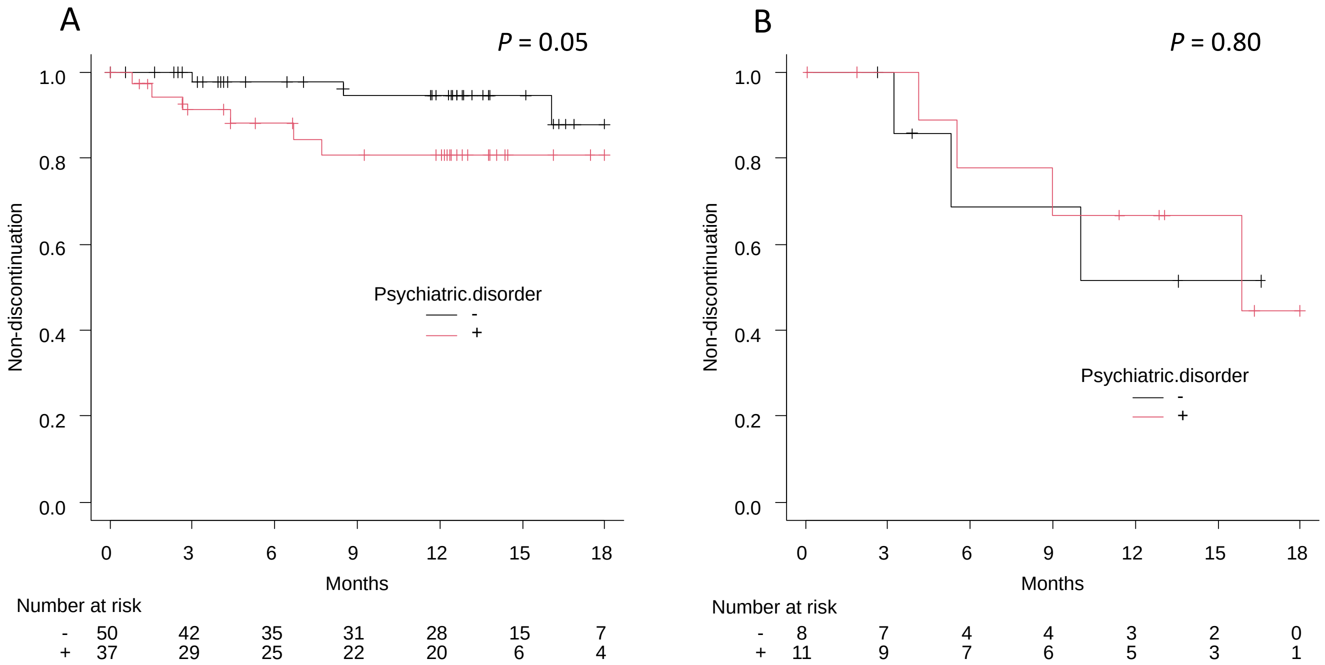

Figure S1. Kaplan–Meier analysis of OA discontinuation in terms of Psychiatric comorbidity [with (red) vs without (black)], stratified by sex.

(A) Males (N=87) **(B)** Females (N=19)

P values were calculated using log-rank test.
